# Protective Effect of Angiotensin 1–7 on Sarcopenia Induced by Chronic Liver Disease in Mice

**DOI:** 10.3390/ijms21113891

**Published:** 2020-05-29

**Authors:** Francisco Aguirre, Johanna Abrigo, Francisco Gonzalez, Andrea Gonzalez, Felipe Simon, Claudio Cabello-Verrugio

**Affiliations:** 1 Laboratory of Muscle Pathology, Fragility and Aging, Department of Biological Sciences, Faculty of Life Sciences, Universidad Andres Bello, Santiago 8370146, Chile; f.aguirregalaz@hotmail.com (F.A.); j.abrigo.leon@gmail.com (J.A.); f.gonzalez.wistuba@gmail.com (F.G.); ap.gonzalezrojas@gmail.com (A.G.); 2 Millennium Institute on Immunology and Immunotherapy, Santiago 8370146, Chile; fsimon@unab.cl; 3 Center for the Development of Nanoscience and Nanotechnology (CEDENNA), Universidad de Santiago de Chile, Santiago 8350709, Chile; 4 Millennium Nucleus of Ion Channels-Associated Diseases (MiNICAD), Universidad de Chile, Santiago 8370146, Chile; 5 Laboratory of Integrative Physiopathology, Department of Biological Science, Faculty of Life Science, Universidad Andres Bello, Santiago 8370146, Chile

**Keywords:** sarcopenia, Angiotensin-(1-7), muscle atrophy, cirrhosis, strength

## Abstract

Sarcopenia associated with chronic liver disease (CLD) is one of the more common extrahepatic features in patients with these pathologies. Among the cellular alterations observed in the muscle tissue under CLD is the decline in the muscle strength and function, as well as the increased fatigue. Morphological changes, such as a decrease in the fiber diameter and transition in the fiber type, are also reported. At the molecular level, sarcopenia for CLD is characterized by: (i) a decrease in the sarcomeric protein, such as myosin heavy chain (MHC); (ii) an increase in the ubiquitin–proteasome system markers, such as atrogin-1/MAFbx1 and MuRF-1/TRIM63; (iii) an increase in autophagy markers, such as LC3II/LC3I ratio. Among the regulators of muscle mass is the renin-angiotensin system (RAS). The non-classical axis of RAS includes the Angiotensin 1–7 [Ang-(1-7)] peptide and its receptor Mas, which in skeletal muscle has anti-atrophic effect in models of muscle wasting induced by immobilization, lipopolysaccharide, myostatin or angiotensin II. In this paper, we evaluated the effect of Ang-(1-7) on the sarcopenia by CLD in a murine model induced by the 5-diethoxycarbonyl-1,4-dihydrocollidine (DDC) hepatotoxin administered through diet. Our results show that Ang-(1-7) administration prevented the decline of the function and strength of muscle and increased the fatigue detected in the DDC-fed mice. Besides, we observed that the decreased fiber diameter and MHC levels, as well as the transition of fiber types, were all abolished by Ang-(1-7) in mice fed with DDC. Finally, Ang-(1-7) can decrease the atrogin-1 and MuRF-1 expression as well as the autophagy marker in mice treated with DDC. Together, our data support the protective role of Ang-(1-7) on the sarcopenia by CLD in mice.

## 1. Introduction

Sarcopenia can be considered as primary (associated with aging) or secondary (associated with chronic diseases, limited mobility or nutritional disorders) [1,2]. In chronic liver disease (CLD), sarcopenia is an essential asset because the loss of muscle strength and muscle mass has been associated directly with CLD and its severity [3,4]. The prevalence of sarcopenia in CLD is high, and it has been shown that there is a loss of muscle mass in the early and late stages of this condition [5,6]. The values depend on the diagnostic technique used, the specific population studied, and gender of the patients [7]. Thus, 60% of patients in the final stages of CLD and 70% of the patients awaiting liver transplantation present sarcopenia, which is in turn associated with decompensation and increased morbidity and mortality [7,8,9]. Sarcopenia is an independent predictor of pre- and post-liver transplantation mortality, with 2.4 times the risk of dying (post-liver transplantation) compared to those without it [10,11]. Specifically, pre-liver transplantation sarcopenia has been associated with higher mortality, a longer stay in the intensive care unit and the hospital, and post-liver transplantation with an increased risk of sepsis and infection [11].

We have studied the mechanisms of sarcopenia by CLD using a murine model [12,13]. In this model, we have reported an imbalance in the muscle mass and the pathways that regulate it. In this context, we have described a transition in the fiber type as well as a decrease in the muscle strength, diameter of fibers and levels of sarcomeric proteins, such as myosin heavy chain (MHC) [12,13,14,15]. Among the intracellular mechanisms, we have observed much activity of the protein degradation pathways in the skeletal muscle. The main protein catabolic pathway is the ubiquitin–proteasome system (UPS) evaluated by the induction of the expression of two muscle-specific E3-ligases atrogin-1 and MuRF-1 [12,13,14,15]. Besides, autophagy is present in cirrhotic muscle showing an increase of LC3II and a decrease of p62 [16,17].

A system capable of regulating the skeletal muscle mass is the Renin-Angiotensin System (RAS) [18]. RAS divides into two axes. The classic pathway, where Angiotensin (Ang) I is converted to Ang II by the Ang-converting enzyme (ACE). Further, Ang II binds to its Ang II type 1 receptor (AT1), which causes a cascade of pathological events that include an increase in fibrosis and protein degradation, as well as a decrease in protein synthesis in the skeletal muscle [18]. The non-classical pathway is represented by Ang-(1-7), the principal peptide of the non-classical pathway of RAS [19]. Ang-(1-7) binds and signals through Mas receptor, which produces benefits for the skeletal muscle, such as antifibrotic and antiatrophic effects [20,21,22]. Besides, Ang-(1-7)/Mas axis prevents the protein degradation pathways activation, such as UPS [20,21,22,23,24].

To our knowledge, to date, no studies are using Ang-(1-7) as a possible therapeutic tool for the management of sarcopenia in CLD. So, the purpose of this research is to determine the effect of the administration of Ang- (1-7) on the functional physical condition (muscle strength and physical performance) and markers of sarcopenia in the skeletal muscle using a murine model of CLD induced by the administration of DDC hepatotoxin in the diet. 

## 2. Results

### 2.1. Ang-(1-7) Prevents a Decline in the Muscle-Dependent Function Induced by Chronic Liver Disease in Mice

**Figure 1:**
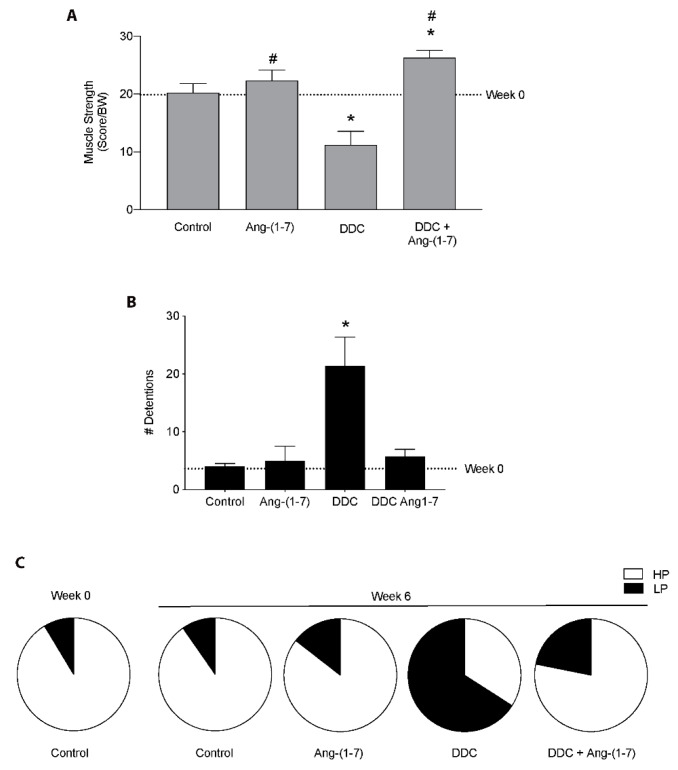
Ang-(1-7) prevents the decrease of muscle function in mice fed with the DDC diet. Male mice C57BL/6J (16 weeks old) were randomly separated into four groups defined as Control, Ang-(1-7), DDC, and DDC + Ang-(1-7). Ang-(1-7) was administered through osmotic pumps. The mice were fed for six weeks with a standard diet or with a DDC-supplemented diet. (**A**) At week 0 and week 6, mice were assessed for muscle strength measured by weightlifting test. Values represent a score which is normalized per body weight. (**B**) Running test results was expressed as the number of detentions in the treadmill. (**C**) Performance in the running test was determined as the time spent in the Low-Performance zone (LP) and High-Performance zone (HP). Values in A, B, and C are shown as mean ± SEM. Five or six animals per group were used for three independent experiments (* *p* < 0.05 vs. Control, # *p* < 0.05 vs. DDC. Two-way ANOVA, Tukey’s multiple comparisons test).

We determined the effect of Ang-(1-7) on the decrease of muscle function in mice fed with the DDC diet. First, we evaluated the muscle strength in vivo measured by the weightlifting test. Figure 1A shows that the decrease of 50% in the muscle strength presented by DDC-treated mice is entirely prevented by Ang-(1-7) [DDC+Ang-(1-7) mice]. Moreover, we can observe that DDC+Ang-(1-7) mice reached a higher strength than control mice. Then, we evaluated the functionality of mice in the treadmill test. In Figure 1B, we observed that the increment in the number of detentions shown by mice fed with a DDC diet in the treadmill test is abolished in the DDC+Ang-(1-7)-treated mice. Besides, Ang-(1-7) also improved the performance of mice in this test. This analysis shows that the Ang-(1-7) administration avoided the high time spent in the low-performance zone shown in the DDC group, showing a similar distribution to control mice (Figure 1C). As a complement to these results, we evaluated the rotarod test in which we observed that Ang-(1-7) abrogated the decrease in the time that DDC-treated mice maintained on the road (Supplementary Figure S1).

Together, these data suggest that Ang-(1-7) improves the muscle function in vivo in the mice with CLD.

### 2.2. Ang-(1-7) Improves the Decrease in Skeletal Muscle Strength and Fatigue in Mice with Chronic Liver Disease

**Figure 2:**
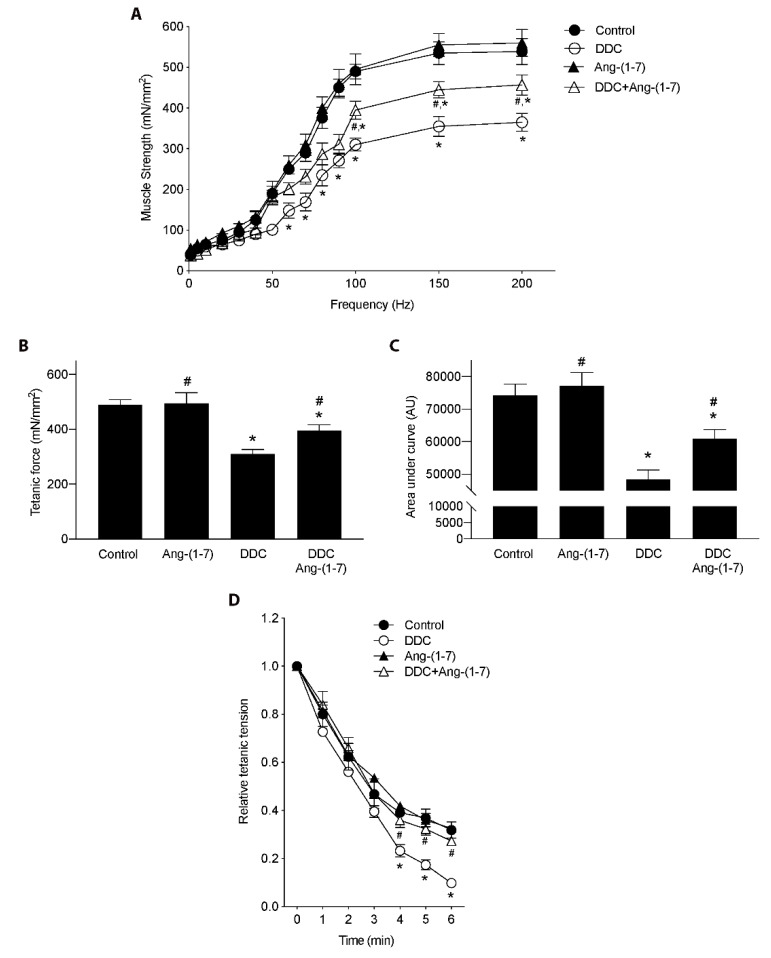
Preventive effect of Ang-(1-7) on the decrease of muscle strength and fatigue induced by DDC diet in gastrocnemius muscle of mice. Male mice C57BL/6J (16 weeks old) were randomly separated into four groups: Control, Ang-(1-7), DDC, and DDC + Ang-(1-7). DDC was administrated by diet for six weeks, whereas Ang-(1-7) was administered through osmotic minipumps for six weeks. Five or six animals per group were used for three independent experiments. At the end of the experiment, gastrocnemius muscle was excised, and electrophysiological assays were performed. (**A**) Muscle strength was measured at different frequencies and represented as mN/mm^2^. (* *p* < 0.05 vs. Control; # *p* < vs. DDC. Two-way ANOVA, Dunnett’s multiple comparisons test). (**B**) Maximal tetanic force in mN/mm^2^. (* *p* < 0.05 vs. Control; # *p* < vs. DDC. One-way ANOVA, Tukey’s multiple comparisons test). (**C**) Muscle strength represented as area under each curve of experiments showed in A. (* *p* < 0.05 vs. Control; # *p* < vs. DDC. One-way ANOVA, Tukey’s multiple comparisons test). (**D**) Fatigue was evaluated as tension relative to tetanic tension for six min. Values are expressed as mean ± SEM. (* *p* < 0.05 vs. Control; # *p* < vs. DDC. Two-way ANOVA, Dunnett’s multiple comparisons test).

Since the administration of Ang-(1-7) improved the performance in the functional tests for living mice, we decided to evaluate if these observations can be extrapolated to muscle strength in isolated gastrocnemius. For that, we performed electrophysiological assays to measure tetanic force. Figure 2A depicts curves of force versus frequency, which shows that Ang-(1-7) partially restored the strength decreased by the DDC diet. These results are corroborated with the analysis of tetanic force (Figure 2B), as well as the analysis of the area under each curve of the figure shown in A (Figure 2C).

Also, we evaluated the fatigue of the gastrocnemius muscles. We determined that strength in the muscle of mice fed with the DDC diet decline faster (more fatigable) than in the muscle of mice fed with the control diet, such as can be observed in 3 to 6 min of the measure (Figure 2D). When the effect of Ang-(1-7) administration to mice fed with DDC [DDC+Ang-(1-7) group] is determined, the kinetic of decline in force is less fatigable than the DDC group and therefore is similar to the control or Ang-(1-7) alone groups (Figure 2D).

Together, these results indicate that Ang-(1-7) can prevent a decrease in muscle strength and higher fatigue of isolated gastrocnemius in mice with sarcopenia by CLD.

### 2.3. The Decreased Fiber Diameter of Gastrocnemius from Mice with CLD is Abrogated by Ang-(1-7)

**Figure 3:**
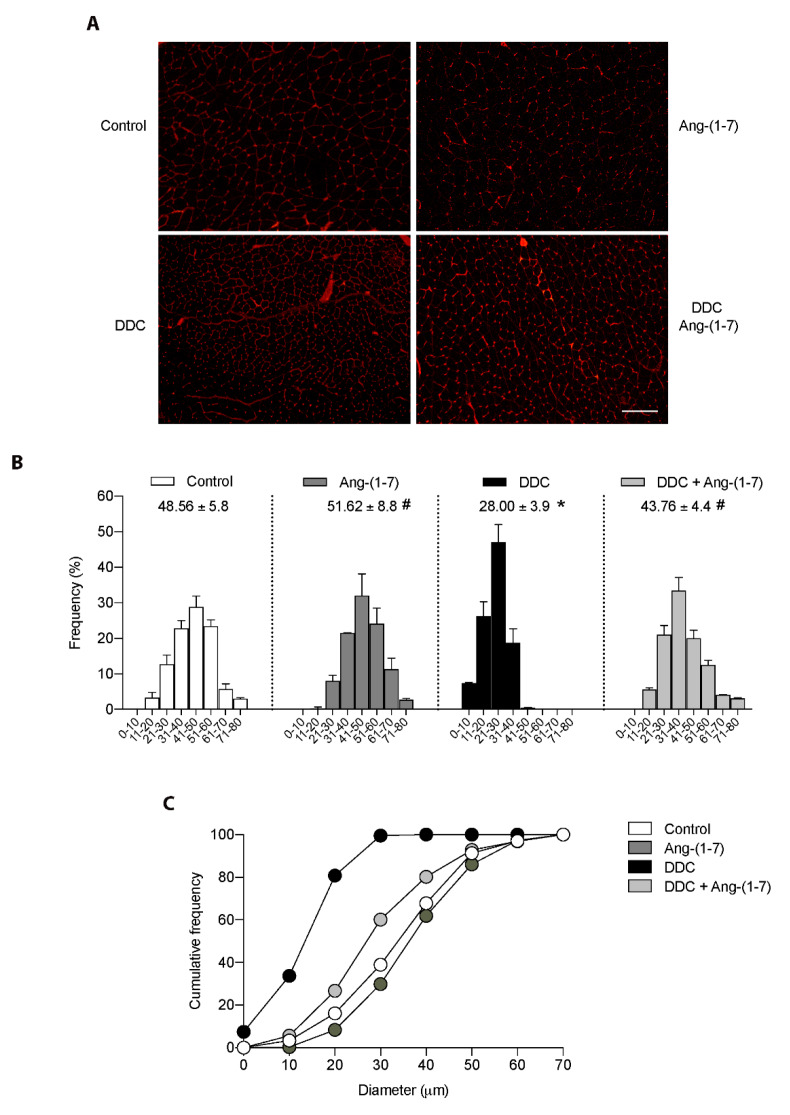
Ang-(1-7) abrogates the diminution of muscle fibers diameter in mice fed with the DDC diet. Male mice C57BL/6J (16 weeks old) were fed with a standard diet (Control) or DDC-supplemented (DDC) for six weeks. Ang-(1-7) was administered for six weeks through osmotic minipumps: The groups were defined as Control, Ang-(1-7), DDC, and DDC + Ang-(1-7). At the end of the experiment, gastrocnemius muscles were excised and frozen. Three independent experiments were performed, using five mice for each experimental condition. (**A**) Cross-sections of gastrocnemius were stained with WGA to delimit muscle fiber sarcolemma. Scale bar: 120 µm. (**B**) Minimal Feret’s diameters were determined in gastrocnemius cross-sections. The values were grouped in ranges from 0 to 80 μm, and frequency per range was plotted as the percentage considering the total amount of quantified fibers. The mean of the diameter per condition is shown. (* *p* < 0.05 vs. Control; # *p* < vs. DDC. One-way ANOVA, Tukey’s multiple comparisons test). (**C**) The data was also represented as a cumulative frequency plot according to the diameter range.

A wheat germ agglutinin (WGA) stain was performed in the gastrocnemius muscle to evaluate the effect of Ang-(1-7) on the decreased fiber diameter in the DDC group. Figure 3A shows that the qualitative decrease in the fiber diameter observed in mice fed the DDC diet showed an evident recovery towards standard fiber diameter after Ang-(1-7) administration [DDC+Ang-(1-7) group]. These observations were quantified as Feret’s diameter in Figure 3B. In this figure, we observed an evident displacement towards smaller sizes on the distribution of fiber abundancy. The same Figure 3B shows the average diameter for the experimental groups. Thus, we observed that the decreased mean of the diameter in DDC-treated mice (28.00 ± 3.9 vs. 48.56 ± 5.8 µm in control mice) was recovered by Ang-(1-7) treatment (43.76 ± 4.4 µm). Besides, the data was analyzed for the parameter of cumulative frequency, indicating that in the DDC group there is > 95 % of the fiber abundancy in the range 0 to 30 µm, which is decreased by Ang-(1-7) to 70.1% (DDC+Ang-(1-7) group), whereas 38.3 and 22.3% is observed in control and Ang-(1-7) alone groups (Figure 3C).

These data indicate that Ang-(1-7) can prevent the morphometric changes, as the fiber diameters in sarcopenia induced by CLD.

### 2.4. Ang-(1-7) Prevented the Transition of Fiber Type in Gastrocnemius from Mice with CLD

**Figure 4:**
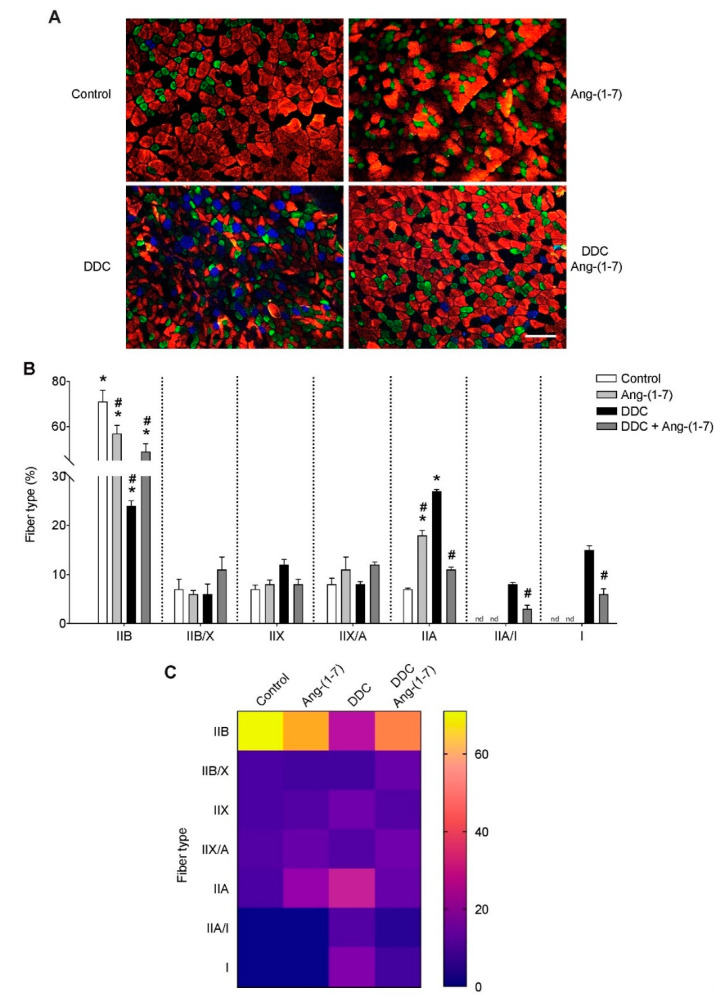
Ang-(1-7) prevents the fiber type transitions in gastrocnemius muscle from mice fed with the DDC diet. Male mice C57BL/6J (16 weeks old) were randomly separated into four groups: Control, Ang-(1-7), DDC, and DDC + Ang-(1-7). DDC was administrated by diet for six weeks, whereas Ang-(1-7) was administered through osmotic minipumps for six weeks. In each experiment, five mice were used for each experimental condition. (**A**) At the end of the experiment, mice were sacrificed, and cryosections of gastrocnemius muscles were obtained. The fiber type was determined through immunofluorescent detection of myosin heavy chain isoforms (IIA, IIB, I). Scale bar: 120 µm. (**B**) Quantitative analysis of the fiber type was plotted and shown as the percentage of fiber types relative to the total fibers per field. Values represent the mean ± SEM. (* *p* < 0.05 vs. Control, # *p* < vs. DDC. (* *p* < 0.05 vs. Control; # *p* < vs. DDC. One-way ANOVA, Tukey’s multiple comparisons test). (**C**) The color map represents the abundance of different fiber types in each condition, from low (blue) to high (yellow) grade.

We have previously described a shift in the fiber type of gastrocnemius muscle in mice fed with the DDC diet, showing an increase of the oxidative type I fibers and a decrease in the glycolytic IIB fibers [12]. Figure 4A shows that this transition observed in the DDC group is partially prevented by Ang-(1-7), showing a phenotype more similar to control mice. In the quantification (Figure 4B), it is possible to note that the administration of Ang-(1-7) to mice treated with DDC [DDC+Ang-(1-7)] partially recovered the decrease of the IIB fiber in DDC group (Control: 71 ± 10.1; DDC: 24 ± 2.1; DDC+Ang-(1-7): 49 ± 7.0). In the case of IIA fibers, Ang-(1-7) also produced a partial shift in DDC-treated mice (Control: 7 ± 0.5; DDC: 27 ± 0.7; DDC+Ang-(1-7): 11 ± 1.0). Besides, it is possible to observe that in the DDC+Ang-(1-7) group, there is a decrease of approximately 50% in the proportion of IIA/I and I fibers compared to the DDC group, maintaining a low grade of oxidative fibers which are not detected in the control and Ang-(1-7) alone groups. These results are represented in the color mapping (Figure 4C).

### 2.5. Decrease of the Myosin Heavy Chain Protein Levels, an Increase of UPS and Autophagy Are Prevented by Ang-(1-7) Administration in Gastrocnemius from Mice with CLD

**Figure 5:**
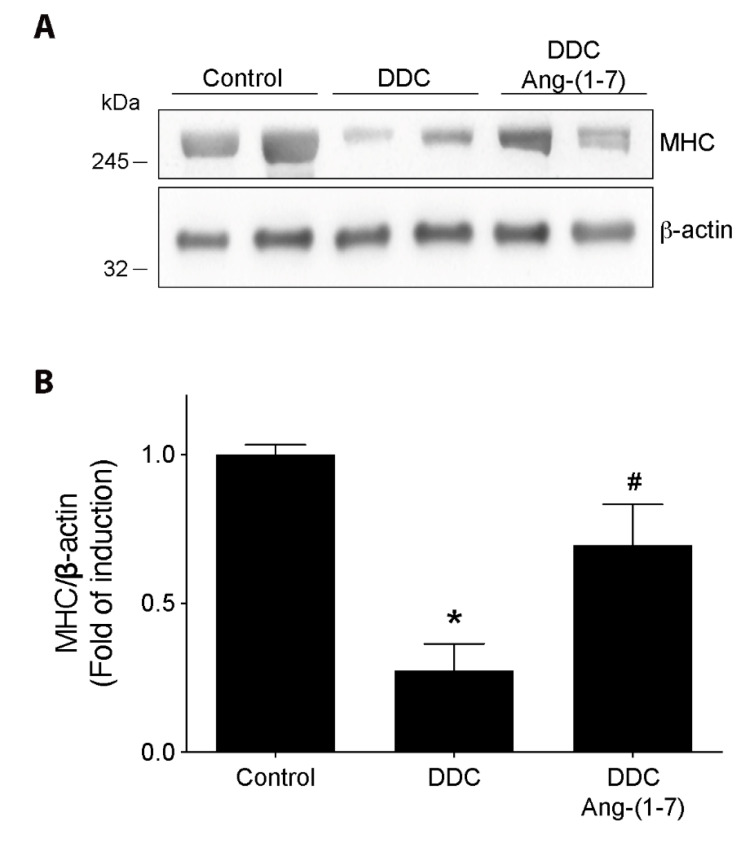
Preventive effect of Ang-(1-7) on decreased myosin heavy chain (MHC) protein levels in gastrocnemius from DDC-treated mice. C57BL/6J male mice were fed for six weeks with a standard diet (Control) or DDC-supplemented diet (DDC) with or without Ang-(1-7) (through osmotic pumps). For three independent experiments, five mice were used for each experimental condition. (**A**) At the end of 6 weeks the mice were sacrificed, gastrocnemius muscle was removed, and protein extraction was performed to detect MHC protein levels by western blot. β-actin was used as the loading control, and molecular weights are depicted in kilodaltons. (**B**) Densitometric analysis of the bands is expressed as fold of induction relative to control. Values represent the mean ± SEM. (* *p* < 0.05 vs. Control, # *p* < vs. DDC. One-way ANOVA, Tukey’s multiple comparisons test).

A parameter directly related to muscle function and strength is the level of sarcomeric proteins. Thus, we evaluated the effect of Ang-(1-7) on the MHC protein levels in the gastrocnemius muscles from mice fed with a DDC diet. Figure 5A shows the MHC levels evaluated by western blot, and Figure 5B shows the quantitative analysis. These figures show that the decrease of MHC levels in the DDC group is partially recovered by Ang-(1-7) administration [Control: 1.0 ± 0.06; DDC: 0.28 ± 0.15; DDC+Ang-(1-7): 0.70 ± 0.24]. Besides, Ang-(1-7) alone does not change the MHC levels compared to the control group (Supplementary Figure S2A,B).

**Figure 6:**
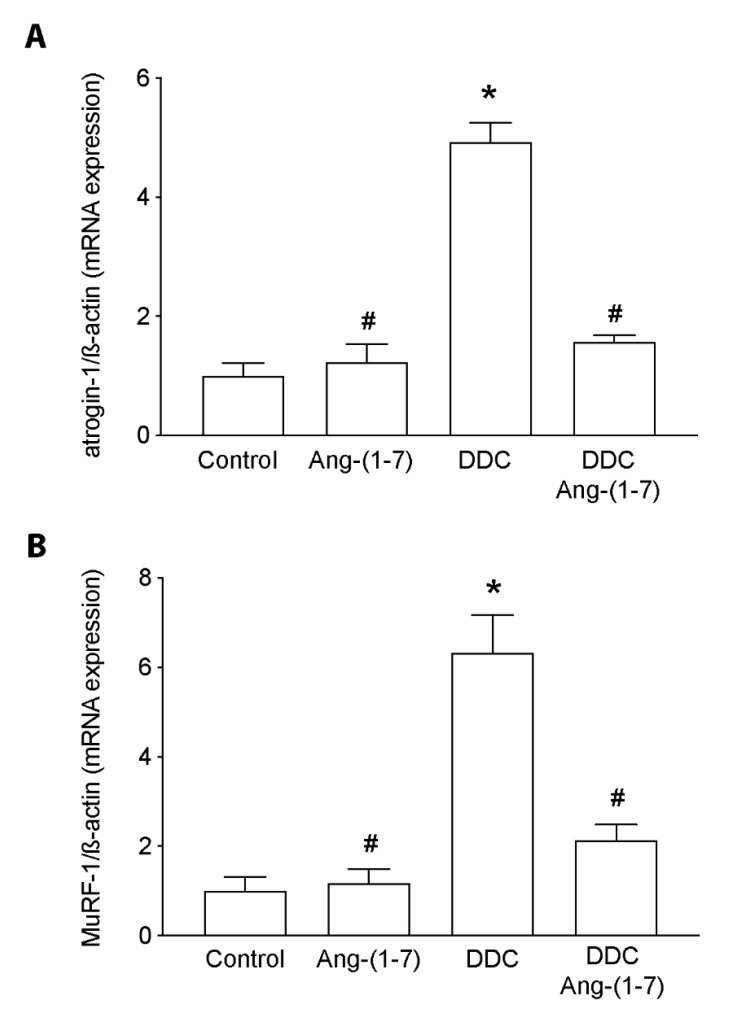
Ang- (1-7) decreases expression levels of *MuRF-1* and *atrogin-1* in gastrocnemius muscle of mice fed with the DDC diet. C57BL/6J male mice were fed with a standard diet (Control) or DDC-supplemented diet (DDC) with or without Ang-(1-7) (through osmotic minipumps). Four animals per group were used for three independent experiments. After six weeks, the mice were sacrificed, and the gastrocnemius muscle was removed. RNA extraction was performed to detect (**A**) *MuRF-1* and (**B**) *atrogin-1* expression by RT-qPCR, using *β-actin* as a housekeeping gene. Values represent the mean ± SEM. (* *p* < 0.05 vs. Control, # *p* < vs. DDC. One-way ANOVA, Tukey’s multiple comparisons test).

The primary process associated with MHC degradation and affecting its levels is the ubiquitin-proteasome system. To evaluate its contribution, we detected the *atrogin-1* and *MuRF-1* expression as well as we evaluated as Ang-(1-7) affects it. We can observe that Ang-(1-7) prevented the increment of the *atrogin-1* (Figure 6A) and *MuRF-1* (Figure 6B) expression induced by the DDC diet being restored to the normal levels (control group).

**Figure 7:**
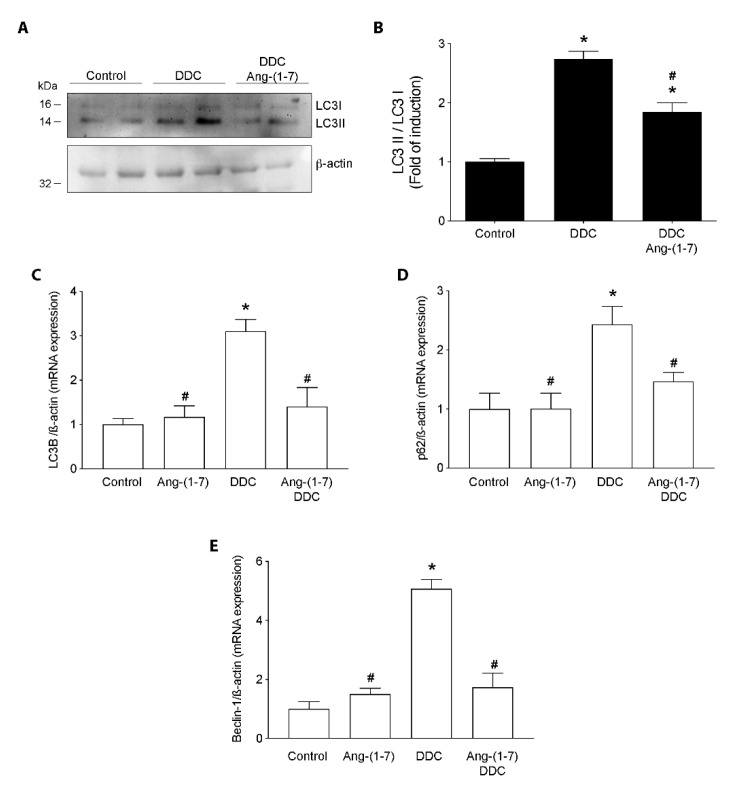
Ang- (1-7) prevents the increment of autophagy in gastrocnemius from DDC-treated mice. C57BL/6J male mice were fed a standard diet (Control) or DDC-supplemented diet (DDC) with or without Ang-(1-7) (through osmotic minipumps). Five animals per group were used for three independent experiments. When the experiment finished (week 6), the gastrocnemius muscle was removed, and RNA and protein extraction were performed. (**A**) LC3 I and LC3 II protein levels were evaluated by western blot. β-actin was used as the loading control. Molecular weights are depicted in kilodaltons. (**B**) Densitometric analysis of the band was performed, and the ratio of LC3 II/LC3 I is shown. Values are expressed as fold of induction relative to control and represent the mean ± SEM. RT-qPCR determined *LC3B* (**C**), *p62* (**D**), and *Beclin-1* (**E**) gene expression. *β-actin* was used as a housekeeping gene (qPCR). Values represent the mean ± SEM. (For B, C, D and E: (* *p* < 0.05 vs. Control, # *p* < vs. DDC. One-way ANOVA, Tukey’s multiple comparisons test).

Another necessary catabolic process associated with sarcopenia by CLD is autophagy. Figure 7A,B show that the increase in the LC3II/LC3I ratio detected by western blot in gastrocnemius from mice fed with the DDC diet is prevented in DDC+Ang-(1-7)-treated mice. Besides, Ang-(1-7) alone did not show changes in the LC3II/LC3I ratio compared to the control group (Supplementary Figure S2C,D). To complement this result, we evaluated the expression of gene involved in the autophagy process, such as *lc3b*, *p62*, and *beclin1*. Figure 7C–E show that the DDC group present higher expressions of *lc3B*, *p62*, and *beclin1*, respectively than the control group. In the same figures, we observed the preventive effect of Ang-(1-7) treatment on *lc3B*, *p62*, and *beclin1* gene expression induced by DDC and maintained this expression in levels similar to the control group.

Together, these results indicate that Ang-(1-7) prevents the induction of UPS and autophagy in sarcopenia by CLD.

## 3. Discussion

This study demonstrates that Ang-(1-7) prevents sarcopenia induced by CLD in a murine model that corresponds to the cholestatic disease caused by the administration of a hepatotoxin [25]. Our main findings include the prevention of several parameters impaired in the muscle from mice fed with a DDC diet: muscle strength and function, fiber type and diameter, levels of MHC, UPS and autophagy activation. 

Regarding the mechanism involved in the CLD-induced sarcopenia, the treatment with Ang-(1-7) induces several improvements, such as we have previously reported in other studies. Thus, the prevention in the muscle strength can be ligated to the abrogated decrease on MHC protein levels in mice fed with the DDC diet and treated with Ang-(1-7). These results are in accordance with the molecular mechanisms involved in protein metabolism [18]. Thus, in this paper, we have reported that UPS members, such as atrogin-1 and MuRF-1, which are directly related to the sarcomeric protein degradation (e.g., MHC), are regulated by Ang-(1-7) administration [18,19]. Several reports indicate that atrogin-1 and MuRF-1 expression are considered atrophic markers. In this context, our results are consistent with the role previously described by us about the anti-atrophic properties of Ang-(1-7), explicitly downregulating the members of UPS. In this line, we also reported here that Ang-(1-7) could decrease the increased muscle autophagy induced by the DDC diet. Interestingly, the DDC diet also caused an increase of *lc3b*, *p62*, and *beclin1* gene expression, probably as a response to maintain the incremented autophagic flux. In this context, Ang-(1-7) prevented not only the autophagy but also the incremented gene expression that help to keep the autophagic process induced by DDC. 

The signaling pathways involved in muscle mass regulation and altered by Ang-(1-7) include proteolytic and anabolic for protein and oxidative stress [18,19]. Even though these pathways were not evaluated in this paper, we have previously described that Ang-(1-7) can downregulate the NF-κB, FoxO and also activate the pathway IGF-1/IGFR/PKB/ mTOR/p70S6K [22,23]. Since the crosstalk reported between both processes, we could speculate that Ang-(1-7) could activate PKB directly through Mas receptor or by induction of IGF-1/IGFR activation. On the one hand, the PKB activation could be responsible for the inhibition of the FoxO family of transcription factors and therefore the inhibition of the MuRF-1 and atrogin-1 gene expression, with a decline in the UPS activity and minor degradation of sarcomeric protein [26,27,28]. On the other hand, PKB activation could be responsible for the increase in protein synthesis through the mTOR-dependent-pathway. Besides, we could also speculate that the activation of mTOR would produce a decrease in the autophagy [29]. Thus, our data are consistent with several lines of evidence indicating crosstalk between the pathways that regulate muscle mass, and that could explain the effects of Ang-(1-7) as an anti-atrophic molecule in skeletal muscle.

Regarding the soluble factors involved in the sarcopenia induced by CLD, there are several candidates. Two of them are myostatin and TNF-α, whose levels are increased during CLD [11,16,30]. Myostatin is a myokine that in high levels produces muscle wasting [31] through a mechanism that involves the Smad pathway and the UPS. Interestingly, we have recently demonstrated that Ang-(1-7) inhibits myostatin-induced muscle wasting [24]. Besides, TGF-β, another member of the same family of proteins, induces muscle atrophy, which is also prevented by Ang-(1-7) [21]. 

In the case of TNF-α, this is a proinflammatory cytokine that induces muscle atrophy [32]. However, to date, direct regulation of Ang-(1-7) on the TNF-α-induced muscle wasting has not been reported. Despite these candidates, other molecules are increased in the plasma during CLD, such as ammonia and bile acids that could regulate the metabolic process in several tissues, including the skeletal muscle.

Several antecedents support the role of Ang- (1-7) in preventing skeletal muscle loss in various conditions of sarcopenia. For the first time, preclinical studies from our group demonstrated the anti-atrophic properties of Ang-(1-7) using models of muscle wasting that are dependent on Ang-II and independent on this peptide (such as muscle atrophy by disuse, LPS, chronic liver disease) [18,22,23,33]. Other studies using models of aging, muscle dysfunction for Alzheimer’s disease, or damage for exercise also have contributed to reinforcing the idea of Ang-(1-7) as a peptide that prevents muscle damage [34,35,36,37,38,39]. 

The clinical use of Ang-(1-7) has the advantage of producing an excellent therapeutical effect without side effects, probably because it is an endogenous peptide. In this line, a study showed that the oral administration of Ang-(1-7) for seven days in humans was sufficient to reduce pain and muscle damage markers after eccentric-overload exercise [40]. The results of this study indicate that the use of Ang- (1-7) is possible in doses that allow positive effects on the human skeletal muscle

In summary, we have demonstrated that Ang-(1-7) prevents CLD-induced sarcopenia in mice.

## 4. Materials and Methods

### 4.1. Animals

Male mice C57BL/6J (16 weeks old) strain was used. The animals were randomized and separated into experimental groups to perform three independent experiments. The mice were fed with two different diets for six weeks: a standard diet and a diet supplemented with 0.1% 5-diethoxycarbonyl-1,4-dihydrocollidine (DDC) (Sigma-Aldrich, St Louis, MO, USA) [12,13]. Ang-(1-7) (Sigma-Aldrich, St Louis, MO, USA) was administered through osmotic minipumps (Alzet-Durect, Cupertino, CA, USA) for six weeks, with a dose of 100 ng/Kg/min as previously was reported [22]. A group with Ang-(1-7) alone was performed (Ang-(1-7)). Thus, the experimental conditions were: Control mice, fed with standard diet; Ang-(1-7) mice, fed with standard diet and treated with Ang-(1-7); DDC mice, fed with DDC-supplemented diet; DDC+Ang-(1-7) mice, fed with DDC diet and treated with Ang-(1-7). Each experimental group is made up of 5 to 6 mice. At the end of the experiment, the animals were euthanized under anesthesia (5% isoflurane). The muscles were dissected, weighed, and rapidly frozen in isopentane and stored at −80 °C until processing. All applicable international, national, and institutional guidelines for the care and use of animals were followed. All procedures performed in studies involving animals had the formal approval of the Animal Ethics Committee at the Universidad Andrés Bello institution.

### 4.2. Weightlifting Test

Before starting the experiment and when it finished, the mice were subjected to a measurement of muscle strength by a weightlifting test. Briefly, the apparatus consisted of a series of chain links of increasing length attached to a ball of tangled fine wire. The number of links ranged from one to seven, with total weights between 15.5 and 54.1 g. Before performing the test, the mice were subjected to training (once per day for a week). To achieve the test, the mouse grasps (with its forepaws) the different weights, and a score was assigned. The final score was calculated as the summation of the product between the link weight and the time held. The mean of three measures from each mouse was normalized by the bodyweight [12,41].

### 4.3. Running Test

Before starting the experiment and when it finished, the animals perform an aerobic exercise test on a treadmill. A conditioning period was performed three times a week, during a week before the test. The experiment consisted of 15 min at 20 cm/s on the tape, divided into three sessions of 5 min, with a rest period of 5 min between each session. For the analysis, the lane distance was split into two zones: zone distant from the starting point (High-Performance or HP), and the region closest to the starting point (Low-Performance or LP). The test was recorded, and the videos were analyzed to calculate the residence time of the mice in each of the two zones described above. Additionally, the detentions of each mouse in the LP zone were counted [12,20].

### 4.4. Rotarod

Before starting the experiment and when it finished, the mice were subjected to an exercise trial in rotarod. For that, the mice were placed in the rotor with an initial rotation speed of 4 rpm. The fuel speed gradually increases to 35 rpm for 5 min, and the total time that the animals manage to stay on the wheel is reached [12].

### 4.5. Isometric Strength and Fatigue of Skeletal Muscle Isolated

When the experiment finished, contractile properties were evaluated in isolated muscles by electrophysiological measurements [12,42,43]. For this, the gastrocnemius was removed and immersed in an oxygenated Krebs-Ringer solution. The maximum length of major contraction (Lo), and the micromanipulation of the muscle length was determined to produce the maximum isometric force. The muscle was subjected to stimuli of different frequencies between 1 and 200 Hz, 450 ms in duration, with 2 min of rest between each stimulus. The specific net force was calculated and normalized by the length of the tibia, expressed as mN/mm^2^). Additionally, the muscle fatigue tests were performed as previously described [44,45]. 

### 4.6. RNA Extraction and cDNA Synthesis

Total RNA was extracted from muscle using Chomczynski’s solution: the muscle was homogenized with ultraturrax, and the resulting lysate was placed in centrifuge tubes. Chloroform was then added to each sample, vigorously stirred by inversion (15 s) and incubated for 15 min at 4 °C. The samples were centrifuged at 16,000× *g* at 4 °C for 15 min, and the aqueous (upper) phase was carefully transferred to a new tube. RNA was precipitated by adding 1 volume of cold isopropanol, which was mixed with the sample and incubate ON at −20 °C. Subsequently, the samples were centrifuged at 16,000× *g* at 4 °C for 15 min, and the supernatant was carefully removed. The pellet was washed twice with 75% ethanol (cold), resuspended in nuclease-free water, and its purity was evaluated by measuring the absorbance at 260 and 280 nm in a spectrophotometer (Abs260/Abs280 between 1.7–2.1 is considered pure). Then, two µg of RNA were transcribed into cDNA, for which the samples were treated with DNAse I (0.1 U/µL), and incubated 15 min at 37 °C, then EDTA (2.5 mM) was added and incubated at 65 °C for 10 min. Subsequently, random primers (1 µg/µL) were added, and the mix was incubated at 70 °C for 5 min. Further, a mixture of dNTP and DTT (final concentrations of 0.75 mM; 0.3 U µL; 1.5 mM respectively) was added to further to be incubated at 25 ° C for 10 min. Finally, the M-MLV reverse transcriptase (1.5 U) was added, and they were subjected to the 10 min PCR cycle at 25 °C, 90 min at 37 °C, 10 min at 70 °C, and a final cycle at 4 °C.

### 4.7. qPCR

cDNA obtained from reverse transcription was analyzed to evaluate the gene expression of *murf-1*, *atrogin-1*, *lc3b*, *p62*, *beclin1*, and *β-actin* genes using SyBR Green. Relative PCR was performed in triplicate using an Eco Real-Time PCR System (Illumina, San Diego, CA, USA). The mRNA expression was calculated using the comparative ΔΔCt method, normalized relative to *β-actin* gene expression [46].

### 4.8. Muscle Fiber’s Diameter Determination and Quantification

Cryosections of 8 µm gastrocnemius muscle were stained with Wheat germ agglutinin (WGA) attached to Alexa-Fluor^®^ 594 (Thermo Fisher Scientific, Waltham, MA, USA). For this, the muscle sections were washed once with 1X HBSS (Hank’s balanced salt solution), and incubated with Alexa-Fluor^®^ 594 WGA (1: 200 diluted in 1X HBSS) for 30 min at 37 °C. Subsequently the cuts were mounted with fluorescent mounting medium (Agilent Dako, Santa Clara, CA, USA). Images were acquired in a Motic BA310 fluorescence microscope (Motic, Hong Kong). The fiber size was determined by calculating the Feret’s diameter using Image J software (NIH, Bethesda, MD, USA) by manual selection of the fibers, and the values were grouped in 5 µm diameter ranges [33].

### 4.9. Determination of Fiber Types in Gastrocnemius

The fiber types in the gastrocnemius muscles were determined by immunofluorescence analysis of MHC isoforms using primary antibodies against the specific types of myosin. Cross-sections (8 µm thick) from gastrocnemius muscle were placed 15 min at room temperature. A wash was performed with 1× PBS, and subsequently, they were incubated for 30 min at room temperature with Mouse-to-Mouse 1:10 (ScyTek Laboratories, Inc., Logan, UT, USA) diluted in PBS. Then, they were blocked for 1 h with PBS-10% goat serum, 1:10 mouse-to-mouse, and incubated with a mixture of primary antibodies against myosin type I (A4.951; Developmental Studies, Hybridoma Bank, University of Iowa, Iowa, IA, USA), myosin type IIa (SC-71; Developmental Studies, Hybridoma Bank, University of Iowa, Iowa, IA, USA) and myosin type IIb (BF-F3; Developmental Studies, Hybridoma Bank, University of Iowa, Iowa, IA, USA). Then, they were washed three times with 1× PBS and incubated for 2 h with a mixture of secondary antibodies diluted in PBS-10% goat serum, 1:10 mouse-to-mouse. Finally, three washes were performed with 1× PBS and were mounted with fluorescent mounting medium (Agilent Dako, Santa Clara, CA, USA) [12,47]. Images were captured in a Leica SP8 confocal microscope. Image analysis was performed using ImageJ software (NIH, Bethesda, MD, USA). The A4.951 hybridoma, a monoclonal antibody developed by Fischman, D.A., also as SC-71 and BF-F3 hybridomas monoclonal antibodies developed by Schiaffino, S. were obtained from the Developmental Studies Hybridoma Bank, created by the NICHD of the NIH and maintained at The University of Iowa, Department of Biology, Iowa City, IA 52242.

### 4.10. Western Blot

For western-blot analysis, 30 to 50 μg of protein were used. The samples were mixed with 60 mM Tris-HCl pH 6.8, SDS, β-mercaptoethanol, and then subjected to SDS-PAGE. They were subsequently transferred to PVDF membranes for 1.5 h. Later, the membranes were blocked for 1 h, and incubated with the corresponding primary antibody: mouse anti-MHC (1:1000 MF-20; Developmental Studies, Hybridoma Bank, University of Iowa, Iowa, IA, USA), rabbit anti- β-actin (1:2000, Abcam, Cambridge, MA, USA), rabbit anti- LC3b (1:1000, Cell Signaling, Danvers, MA, USA). After the incubation time, the membranes were washed and incubated with the corresponding secondary antibody. Finally, the binding of the primary antibody / secondary antibody complex to the protein samples was detected using the chemiluminescence procedure through an image documentation system, Fotodyne (Fisher Scientific, St. Waltham, MA, USA). Densitometric analysis of the bands was performed using ImageJ software (NIH, Bethesda, MD, USA), and standardized relative to the respective load control of each sample. The MF-20 hybridoma, a monoclonal antibody developed by Fischman, D.A., was obtained from the Developmental Studies Hybridoma Bank, created by the NICHD of the NIH and maintained at The University of Iowa, Department of Biology, Iowa City, IA 52242.

### 4.11. Statistical Analysis

The statistical analysis of the data was performed with the Prism 8.0 analysis software (GraphPad Software, San Diego, CA, USA). The normality of the data was determined. Data were analyzed with a one, or two-way ANOVA was used as appropriate, with a Dunnett or Tukey post-hoc test. Differences were considered significant when *p* < 0.05.

## Supplementary Materials

The following are available online at https://www.mdpi.com/1422-0067/21/11/3891/s1. Figure S1. Ang-(1-7) improves the muscle function in mice fed with a DDC diet. Male mice C57BL/6J (16 weeks old) were randomly separated into four groups: Control, Ang-(1-7), DDC, and DDC + Ang-(1-7). DDC was administrated by diet for six weeks, whereas Ang-(1-7) was administered through osmotic minipumps for six weeks. A rotarod test evaluated muscle function. Time (in s) that the animal is on the cylinder was registered. The values correspond to the mean ± SEM (five or six animals per group, three independent experiments; (* *p* < 0.05 vs. Control, # *p* < vs. DDC. One-way ANOVA, Tukey’s multiple comparisons test). Figure S2. Ang-(1-7) alone does not produce alterations in myofibrillar proteins or autophagy markers. C57BL/6J male mice were fed for six weeks with a standard diet and separated into two groups: without (Control) or with Ang-(1-7) (administered through osmotic pumps). At the end of 6 weeks, the mice were sacrificed, the gastrocnemius muscle was removed, and protein extraction was performed. (A) MHC and (C) LC3 protein levels were evaluated by western-blot, using β-actin as the loading control, and the molecular weight is depicted in kilodaltons. Densitometric analysis of the bands was performed and shown as fold of induction in MHC (B) and the ratio of LC3II/LC3I (D). Values represent the mean ± SEM of three independent experiments. In each experiment, five mice were used for each experiment.
