# Supplementary material for: Protective Effect of Angiotensin 1–7 on Sarcopenia Induced by Chronic Liver Disease in Mice
Source: Int J Mol Sci. 2020 May 29;21(11):3891. doi: 10.3390/ijms21113891 (PMC7312494; doi:10.3390/ijms21113891)
Supplement: Supplementary file 1 [file ijms-21-03891-s001.pdf]

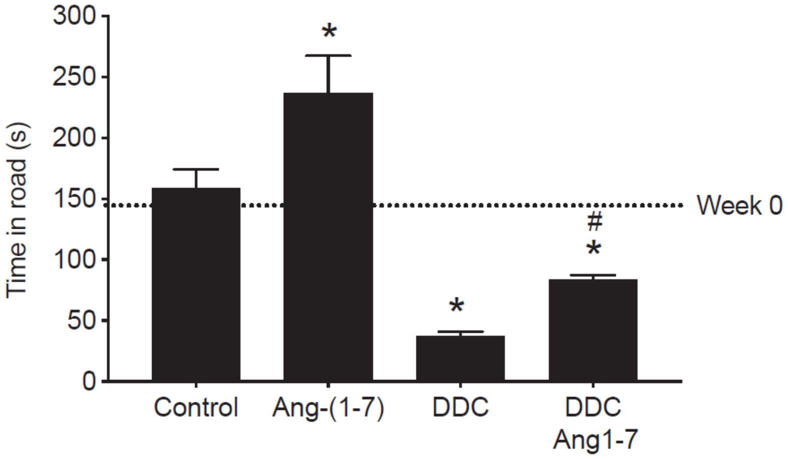

Supplementary Figure S1. Ang-(1-7) improves the muscle function in mice fed with a DDC diet. Male mice C57BL/6J (16 weeks old) were randomly separated into four groups: Control, Ang-(1-7), DDC, and DDC + Ang-(1-7). DDC was administrated by diet for six weeks, whereas Ang-(1-7) was administered through osmotic minipumps for six weeks. A rotarod test evaluated muscle function. Time (in s) that the animal is on the cylinder was registered. The values correspond to the mean ± SEM (five or six animals per group, three independent experiments; (* $p < 0.05$ vs. Control, # $p <$ vs. DDC. One-way ANOVA, Tukey's multiple comparisons test).

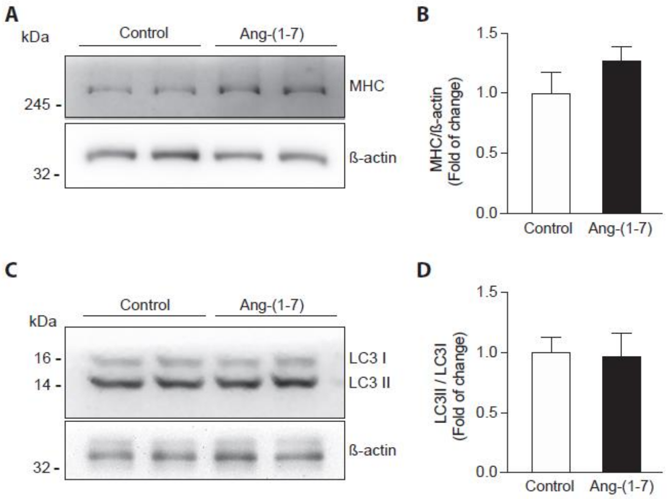

Supplementary Figure S2. Ang- (1-7) alone does not produce alterations in myofibrillar proteins or autophagy markers. C57BL/6J male mice were fed for six weeks with a standard diet and separated into two groups: without (Control) or with Ang-(1-7) (administered through osmotic pumps). At the end of 6 weeks, the mice were sacrificed, the gastrocnemius muscle was removed, and protein extraction was performed. (**A**) MHC and (**C**) LC3 protein levels were evaluated by western-blot, using β-actin as the loading control, and the molecular weight is depicted in kilodaltons. Densitometric analysis of the bands was performed and shown as fold of induction in MHC (**B**) and the ratio of LC3II/LC3I (**D**). Values represent the mean ± SEM of three independent experiments. In each experiment, five mice were used for each experiment.
